# Crystal structure of 5,11-di­hydro­pyrido[2,3-*b*][1,4]benzodiazepin-6-one

**DOI:** 10.1107/S2056989015006817

**Published:** 2015-04-11

**Authors:** Noura M. Riad, Darius P. Zlotos, Ulrike Holzgrabe

**Affiliations:** a The German University in Cairo, Department of Pharmaceutical Chemistry, New Cairo City, 11835 Cairo, Egypt; b Institute of Pharmacy and Food Chemistry, Wuerzburg University, 97074 Wuerzburg, Germany

**Keywords:** crystal structure, pyridobenzodiazepine, boat conformation, hydrogen bonding

## Abstract

The title compound, C_12_H_9_N_3_O, is an inter­mediate in the synthesis of the muscarinic M2 receptor antagonist AFDX-384. The seven-membered ring adopts a boat conformation and the dihedral angle between the planes of the aromatic rings is 41.51 (9)°. In the crystal, mol­ecules are linked into [001] chains of alternating inversion dimers formed by pairs of N—H⋯O hydrogen bonds and pairs of N—H⋯N hydrogen bonds. In both cases, *R*_2_^2^(8) loops are generated.

## Related literature   

For the synthesis of the title compound, see: Holzgrabe & Heller (2003 ▸). For the biological activity of substituted 5,11-di­hydro­pyrido[2,3-*b*][1,4]benzodiazepin-6-ones, see: Mohr *et al.* (2004 ▸); Tahtaoui *et al.* (2004 ▸).
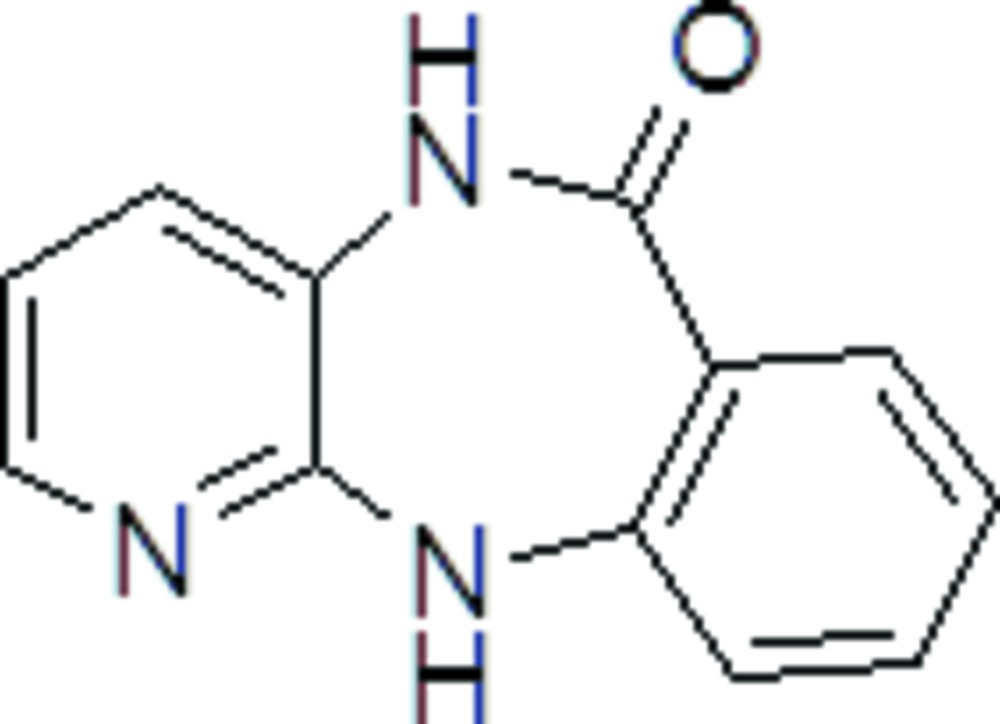


## Experimental   

### Crystal data  

C_12_H_9_N_3_O
*M* *_r_* = 211.22
Triclinic, 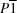

*a* = 3.7598 (5) Å
*b* = 10.2467 (14) Å
*c* = 12.8768 (17) Å
α = 104.628 (6)°
β = 96.616 (5)°
γ = 98.009 (4)°
*V* = 469.43 (11) Å^3^
*Z* = 2
Mo *K*α radiation
μ = 0.10 mm^−1^
*T* = 100 K
0.35 × 0.26 × 0.06 mm

### Data collection  

Bruker APEXII CCD diffractometer
Absorption correction: multi-scan (*SADABS*; Bruker, 2013 ▸) *T*_min_ = 0.898, *T*_max_ = 0.959
6425 measured reflections
2000 independent reflections
1467 reflections with *I* > 2σ(*I*)
*R*_int_ = 0.035

### Refinement  

*R*[*F* ^2^ > 2σ(*F* ^2^)] = 0.041
*wR*(*F* ^2^) = 0.110
*S* = 1.06
2000 reflections
153 parameters
H atoms treated by a mixture of independent and constrained refinement
Δρ_max_ = 0.23 e Å^−3^
Δρ_min_ = −0.22 e Å^−3^

### 

Data collection: *APEX2* (Bruker, 2013 ▸); cell refinement: *SAINT* (Bruker, 2013 ▸); data reduction: *SAINT*; program(s) used to solve structure: *OLEX2*.solve (Bourhis *et al.*, 2015 ▸); program(s) used to refine structure: *SHELXL97* (Sheldrick, 2008 ▸); molecular graphics: *OLEX2* (Dolomanov *et al.*, 2009 ▸); software used to prepare material for publication: *OLEX2*, *Mercury* (Macrae *et al.*, 2006 ▸) and *enCIFer* (Allen *et al.*, 2004 ▸).

## Supplementary Material

Crystal structure: contains datablock(s) I. DOI: 10.1107/S2056989015006817/hb7396sup1.cif

Structure factors: contains datablock(s) I. DOI: 10.1107/S2056989015006817/hb7396Isup2.hkl

Supporting information file. DOI: 10.1107/S2056989015006817/hb7396Isup3.cml

**Fig. 1.**
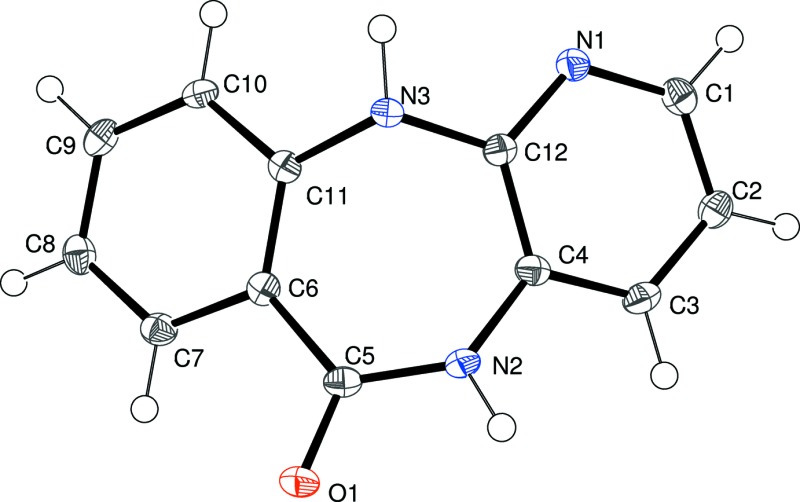
ORTEP drawing of the title compound showing atom labeling and 50% probability displacement ellipsoids.

. DOI: 10.1107/S2056989015006817/hb7396fig1.tif
ORTEP drawing of the title compound showing atom labeling and 50% probability displacement ellipsoids.

**Fig. 2.**
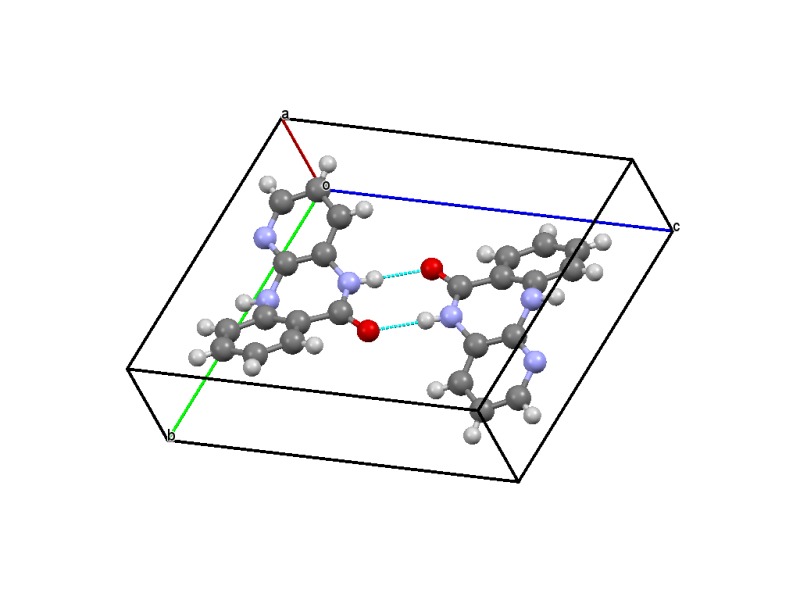
Unit-cell packing of the title compound showing two inverted molecules linked by hydrogen bonds indicated as dotted lines.

. DOI: 10.1107/S2056989015006817/hb7396fig2.tif
Unit-cell packing of the title compound showing two inverted molecules linked by hydrogen bonds indicated as dotted lines.

CCDC reference: 1024195

Additional supporting information:  crystallographic information; 3D view; checkCIF report

## Figures and Tables

**Table 1:** Hydrogen-bond geometry (, )

*D*H*A*	*D*H	H*A*	*D* *A*	*D*H*A*
N2H2O1^i^	0.87(2)	1.98(2)	2.840(2)	175(2)
N3H3N1^ii^	0.93(2)	2.28(2)	3.200(2)	168.7(19)

Symmetry codes: (i) 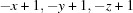; (ii) 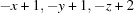.

## supplementary crystallographic information

### S1. Experimental

The title compound was synthesized as previously reported (Holzgrabe & Heller, 2003) and recrystallized from methanol–toluene.

### S2. Refinement

The N- and C-bound H atoms were included in calculated positions and refined as riding: N2—H = 0.86 Å, C—H and N3—H = 0.93 Å with *U*_iso_(H) = 1.2*U*_eq_(C).

### Crystal data

**Table:**

C_12_H_9_N_3_O	*Z* = 2
*M**_r_* = 211.22	*F*(000) = 220
Triclinic, *P*1	*D*_x_ = 1.494 Mg m^−^^3^
*a* = 3.7598 (5) Å	Mo *K*α radiation, λ = 0.71073 Å
*b* = 10.2467 (14) Å	Cell parameters from 1512 reflections
*c* = 12.8768 (17) Å	θ = 2.3–26.2°
α = 104.628 (6)°	µ = 0.10 mm^−^^1^
β = 96.616 (5)°	*T* = 100 K
γ = 98.009 (4)°	Plate, colourless
*V* = 469.43 (11) Å^3^	0.35 × 0.26 × 0.06 mm

### Data collection

**Table:**

Bruker APEXII CCD diffractometer	1467 reflections with *I* > 2σ(*I*)
φ and ω scans	*R*_int_ = 0.035
Absorption correction: multi-scan (*SADABS*; Bruker, 2013)	θ_max_ = 26.8°, θ_min_ = 1.7°
*T*_min_ = 0.898, *T*_max_ = 0.959	*h* = −4→4
6425 measured reflections	*k* = −12→12
2000 independent reflections	*l* = −16→16

### Refinement

**Table:**

Refinement on *F*^2^	Primary atom site location: iterative
Least-squares matrix: full	Hydrogen site location: mixed
*R*[*F*^2^ > 2σ(*F*^2^)] = 0.041	H atoms treated by a mixture of independent and constrained refinement
*wR*(*F*^2^) = 0.110	*w* = 1/[σ^2^(*F*_o_^2^) + (0.0437*P*)^2^ + 0.2092*P*] where *P* = (*F*_o_^2^ + 2*F*_c_^2^)/3
*S* = 1.06	(Δ/σ)_max_ < 0.001
2000 reflections	Δρ_max_ = 0.23 e Å^−^^3^
153 parameters	Δρ_min_ = −0.22 e Å^−^^3^
0 restraints	

### Special details

**Table:**

Experimental. Absorption correctiuon: SADABS-2012/1 (Bruker,2012) was used for absorption correction. wR2(int) was 0.0475 before and 0.0419 after correction. The Ratio of minimum to maximum transmission is 0.9367. The λ/2 correction factor is 0.0015.
Geometry. All e.s.d.'s (except the e.s.d. in the dihedral angle between two l.s. planes) are estimated using the full covariance matrix. The cell e.s.d.'s are taken into account individually in the estimation of e.s.d.'s in distances, angles and torsion angles; correlations between e.s.d.'s in cell parameters are only used when they are defined by crystal symmetry. An approximate (isotropic) treatment of cell e.s.d.'s is used for estimating e.s.d.'s involving l.s. planes.

### Fractional atomic coordinates and isotropic or equivalent isotropic displacement parameters (Å^2^)

**Table:**

	*x*	*y*	*z*	*U*_iso_*/*U*_eq_	
O1	0.3371 (4)	0.33046 (14)	0.49729 (10)	0.0196 (3)	
N3	0.4460 (4)	0.42582 (15)	0.83790 (13)	0.0152 (4)	
N1	0.3852 (4)	0.64248 (15)	0.93418 (12)	0.0157 (4)	
N2	0.3945 (4)	0.51349 (17)	0.63963 (13)	0.0163 (4)	
C4	0.3379 (5)	0.59565 (19)	0.74023 (14)	0.0143 (4)	
C12	0.3836 (5)	0.55643 (19)	0.83732 (14)	0.0133 (4)	
C1	0.3340 (5)	0.7706 (2)	0.93750 (16)	0.0174 (4)	
H1	0.3431	0.8320	1.0051	0.021*	
C10	0.0818 (5)	0.20571 (19)	0.81375 (15)	0.0148 (4)	
H10	0.1291	0.2203	0.8887	0.018*	
C3	0.2728 (5)	0.72576 (19)	0.74596 (15)	0.0168 (4)	
H3A	0.2314	0.7535	0.6829	0.020*	
C6	0.1477 (5)	0.28402 (18)	0.65494 (14)	0.0137 (4)	
C7	−0.0602 (5)	0.15952 (19)	0.59213 (15)	0.0162 (4)	
H7	−0.1071	0.1434	0.5171	0.019*	
C11	0.2231 (5)	0.30746 (18)	0.76774 (14)	0.0130 (4)	
C5	0.2998 (5)	0.37759 (19)	0.59331 (14)	0.0150 (4)	
C2	0.2691 (5)	0.81567 (19)	0.84655 (15)	0.0170 (4)	
H2A	0.2239	0.9039	0.8521	0.020*	
C8	−0.1979 (5)	0.0597 (2)	0.63858 (16)	0.0177 (4)	
H8	−0.3366	−0.0226	0.5956	0.021*	
C9	−0.1254 (5)	0.08462 (19)	0.75055 (15)	0.0168 (4)	
H9	−0.2180	0.0187	0.7831	0.020*	
H3	0.497 (6)	0.419 (2)	0.9087 (19)	0.027 (6)*	
H2	0.467 (6)	0.557 (2)	0.5943 (19)	0.031 (7)*	

### Atomic displacement parameters (Å^2^)

**Table:**

	*U*^11^	*U*^22^	*U*^33^	*U*^12^	*U*^13^	*U*^23^
O1	0.0270 (8)	0.0201 (7)	0.0118 (7)	0.0026 (6)	0.0058 (6)	0.0042 (6)
N3	0.0192 (9)	0.0143 (8)	0.0113 (8)	0.0023 (7)	−0.0008 (7)	0.0036 (7)
N1	0.0174 (8)	0.0157 (8)	0.0137 (8)	0.0016 (7)	0.0032 (6)	0.0039 (7)
N2	0.0212 (9)	0.0165 (9)	0.0122 (8)	0.0010 (7)	0.0043 (7)	0.0062 (7)
C4	0.0119 (9)	0.0174 (10)	0.0125 (9)	−0.0007 (8)	0.0012 (7)	0.0041 (8)
C12	0.0101 (9)	0.0155 (10)	0.0143 (9)	−0.0002 (7)	0.0015 (7)	0.0053 (8)
C1	0.0159 (10)	0.0178 (10)	0.0173 (10)	0.0024 (8)	0.0046 (8)	0.0018 (8)
C10	0.0158 (9)	0.0184 (10)	0.0128 (9)	0.0052 (8)	0.0031 (7)	0.0072 (8)
C3	0.0153 (10)	0.0203 (10)	0.0166 (10)	0.0021 (8)	0.0011 (8)	0.0097 (8)
C6	0.0117 (9)	0.0151 (10)	0.0154 (9)	0.0038 (8)	0.0046 (7)	0.0043 (8)
C7	0.0150 (10)	0.0192 (10)	0.0145 (9)	0.0047 (8)	0.0005 (7)	0.0044 (8)
C11	0.0105 (9)	0.0148 (9)	0.0139 (9)	0.0040 (7)	0.0027 (7)	0.0032 (8)
C5	0.0135 (9)	0.0193 (10)	0.0127 (9)	0.0029 (8)	0.0013 (7)	0.0056 (8)
C2	0.0160 (10)	0.0163 (10)	0.0209 (10)	0.0047 (8)	0.0045 (8)	0.0071 (9)
C8	0.0139 (10)	0.0151 (10)	0.0215 (10)	0.0012 (8)	0.0004 (8)	0.0024 (8)
C9	0.0135 (9)	0.0175 (10)	0.0229 (11)	0.0039 (8)	0.0054 (8)	0.0102 (9)

### Geometric parameters (Å, º)

**Table:**

O1—C5	1.240 (2)	C10—C11	1.396 (3)
N3—C12	1.392 (2)	C10—C9	1.372 (3)
N3—C11	1.406 (2)	C3—H3A	0.9300
N3—H3	0.93 (2)	C3—C2	1.390 (3)
N1—C12	1.332 (2)	C6—C7	1.396 (3)
N1—C1	1.344 (2)	C6—C11	1.399 (2)
N2—C4	1.412 (2)	C6—C5	1.487 (3)
N2—C5	1.347 (2)	C7—H7	0.9300
N2—H2	0.87 (3)	C7—C8	1.380 (3)
C4—C12	1.406 (3)	C2—H2A	0.9300
C4—C3	1.374 (3)	C8—H8	0.9300
C1—H1	0.9300	C8—C9	1.387 (3)
C1—C2	1.372 (3)	C9—H9	0.9300
C10—H10	0.9300		
			
C12—N3—C11	121.58 (15)	C7—C6—C11	119.17 (17)
C12—N3—H3	110.9 (13)	C7—C6—C5	115.70 (16)
C11—N3—H3	112.7 (13)	C11—C6—C5	124.91 (17)
C12—N1—C1	117.87 (16)	C6—C7—H7	119.2
C4—N2—H2	115.9 (15)	C8—C7—C6	121.68 (18)
C5—N2—C4	130.98 (17)	C8—C7—H7	119.2
C5—N2—H2	112.2 (15)	C10—C11—N3	117.55 (16)
C12—C4—N2	123.05 (17)	C10—C11—C6	118.58 (17)
C3—C4—N2	118.46 (17)	C6—C11—N3	123.83 (17)
C3—C4—C12	118.12 (17)	O1—C5—N2	119.17 (17)
N3—C12—C4	121.47 (16)	O1—C5—C6	119.73 (17)
N1—C12—N3	115.93 (16)	N2—C5—C6	121.09 (16)
N1—C12—C4	122.55 (17)	C1—C2—C3	118.16 (18)
N1—C1—H1	118.2	C1—C2—H2A	120.9
N1—C1—C2	123.52 (18)	C3—C2—H2A	120.9
C2—C1—H1	118.2	C7—C8—H8	120.7
C11—C10—H10	119.4	C7—C8—C9	118.69 (18)
C9—C10—H10	119.4	C9—C8—H8	120.7
C9—C10—C11	121.27 (17)	C10—C9—C8	120.60 (18)
C4—C3—H3A	120.2	C10—C9—H9	119.7
C4—C3—C2	119.64 (17)	C8—C9—H9	119.7
C2—C3—H3A	120.2		
			
N1—C1—C2—C3	−3.0 (3)	C7—C6—C11—C10	−1.0 (3)
N2—C4—C12—N3	−7.9 (3)	C7—C6—C5—O1	−22.0 (3)
N2—C4—C12—N1	169.29 (18)	C7—C6—C5—N2	157.23 (17)
N2—C4—C3—C2	−170.60 (17)	C7—C8—C9—C10	−0.5 (3)
C4—N2—C5—O1	170.53 (18)	C11—N3—C12—N1	132.19 (18)
C4—N2—C5—C6	−8.7 (3)	C11—N3—C12—C4	−50.5 (2)
C4—C3—C2—C1	0.4 (3)	C11—C10—C9—C8	0.5 (3)
C12—N3—C11—C10	−129.09 (19)	C11—C6—C7—C8	1.0 (3)
C12—N3—C11—C6	53.5 (2)	C11—C6—C5—O1	152.47 (18)
C12—N1—C1—C2	2.1 (3)	C11—C6—C5—N2	−28.3 (3)
C12—C4—C3—C2	2.7 (3)	C5—N2—C4—C12	42.4 (3)
C1—N1—C12—N3	178.56 (16)	C5—N2—C4—C3	−144.7 (2)
C1—N1—C12—C4	1.3 (3)	C5—C6—C7—C8	175.82 (17)
C3—C4—C12—N3	179.21 (17)	C5—C6—C11—N3	2.0 (3)
C3—C4—C12—N1	−3.6 (3)	C5—C6—C11—C10	−175.34 (17)
C6—C7—C8—C9	−0.2 (3)	C9—C10—C11—N3	−177.23 (16)
C7—C6—C11—N3	176.35 (17)	C9—C10—C11—C6	0.3 (3)

### Hydrogen-bond geometry (Å, º)

**Table:**

*D*—H···*A*	*D*—H	H···*A*	*D*···*A*	*D*—H···*A*
N2—H2···O1^i^	0.87 (2)	1.98 (2)	2.840 (2)	175 (2)
N3—H3···N1^ii^	0.93 (2)	2.28 (2)	3.200 (2)	168.7 (19)

Symmetry codes: (i) −*x*+1, −*y*+1, −*z*+1; (ii) −*x*+1, −*y*+1, −*z*+2.
