# Supplementary figures and images for: Crystal structure of 5,11-di­hydro­pyrido[2,3-b][1,4]benzodiazepin-6-one
Source: Acta Crystallogr E Crystallogr Commun. 2015 Apr 11;71(Pt 5):o304–5. doi: 10.1107/S2056989015006817 (PMC4420050; doi:10.1107/S2056989015006817)

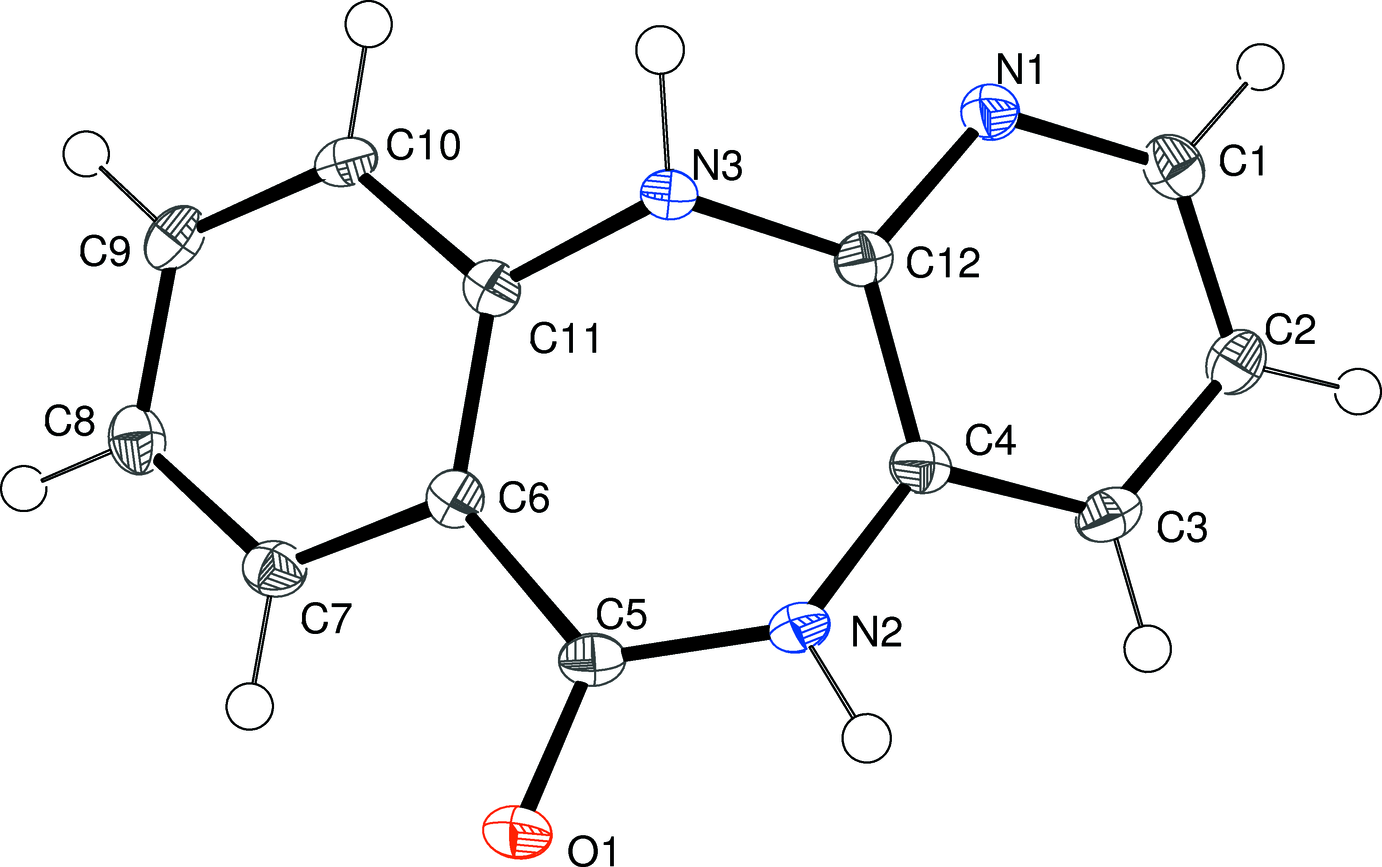

Supplement: Supplementary file 4 [file e-71-0o304-fig1.tif]

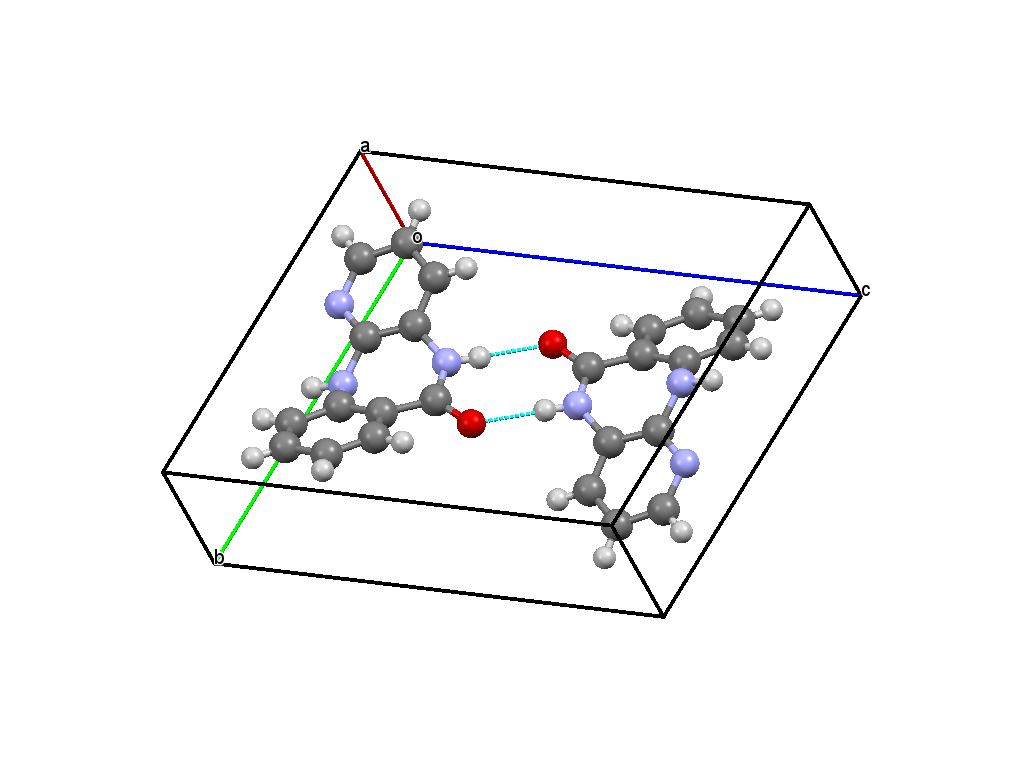

Supplement: Supplementary file 5 [file e-71-0o304-fig2.tif]
